# The Story of the Insane from Year to Year

**Published:** 1902-07-19

**Authors:** 


### THE STORY OF THE INSANE FROM YEAR TO YEAR.

Fourteenth Annual Series.

### THREE COUNTIES ASYLUM AT ARLESEY, BEDS.

At this asylum the numbers fell from 1,035 to 935 during the year, but this decrease was not owing to any lack of lunatics, but to the removal of patients to their own asylum at Hill End. There were 214 first admissions, 48 readmissions, 106 recoveries, and 137 deaths. The average number resident was 974, and the total number under treatment was 1,335. Calculated on the admissions the recoveries stand at 35 per cent., and calculated on the average number resi dent the deaths stand at 14 per cent., the former being under and the latter considerably over the County Asylum average, and also over the Three Counties' average for 30 years. Of the deaths 51 were due to cerebral and spinal diseases, 29 to consumption, 7 to pneumonia, 3 to bronchitis, and 1 to enteritis. Of the year's admissions 47 owed their insanity to various moral causes. Intemperance in drink accounted for 37, and hereditary influence for 64. Dr. De Lisle has had a somewhat unenviable experience for he had three fires during the year, two of these being in the asylum and one in the stackyard. The Asylum Fire Brigade was sufficient to cope with these outbreaks, but in one case damage to the extent of £300 was done. A murderous assault was made by one of the patients on Dr. Munn, the junior assistant medical officer, the weapon used being a piece of steel, part of a corset. The patient was very properly handed over to the police, and it is to be hoped he is now at Broadmoor. The visiting committee say that "the management of the asylum has been carried out by Dr. De Lisle in a very efficient manner." The average cost per head per week was 10s.

### LANCASHIRE ASYLUM, PRESTWICH.

There were 2,692 patients in residence on January 1st, and by December 31st the number had risen to 2,714. There were 378 first admissions, 70 readmissions, 183 discharged recovered, 85 discharged relieved, and 157 deaths. The average number resident was 2,679, and the total number under treatment was 3,128. Calculated on the admissions, the recoveries stand at 41·18 per cent., and the deaths, calculated on the average number resident, stand at 5 90 per cent. The former percentage is above the average of county asylums, and the latter is much below it. Of the deaths 50 were caused by cerebral and spinal diseases, 34 by consumption, 31 by pneumonia, and 6 by other lung diseases. Of the admissions 136 are supposed to owe their insanity to such moral causes as domestic troubles, adverse circumstances, mental anxiety and religion, while of the physical causes we have intemperance in drink heading the list with 137, and hereditary influence with 76. Mr. Ley has the following very sensible remarks on criminal patients: "These persons are in every way most undesirable inmates of an asylum, as their criminal instincts usually become accentuated by their insanity, and their presence in the asylum is a standing menace to order and discipline." The association of criminal lunatics with ordinary patients in our county asylums is an injustice which has been many a time pointed out by visiting committees and by medical superintendents, and it is much to be desired that Parliament could be induced to take notice of the injustice. There are 8 assistant medical officers in this asylum, with salaries ranging from £125 to £300 a year. It does not appear that any of these posts are held by ladies, which we think is a mistake. The cost of maintenance is 9s. 4½d. per head per week.

### LANARK DISTRICT ASYLUM AT HARTWOOD.

At the end of the year this asylum contained 700 patients. There were 147 first admissions, 58 readmissions, 101 recoveries, 23 discharged relieved, and 47 deaths. The average number resident was 690, and the total number under treatment was 874. Calculated in the usual way the recoveries stand at 49·3 per cent., and the deaths at 6·8 per cent. Of the deaths 15 were caused by cerebral and spinal diseases, and 5 by consumption. Of the year's admissions the insanity was caused by moral causes in 18 instances, by intemperance in drink in 40, by hereditary influence in 17, and by injury to head in 4. Dr. Campbell Clark's report is very short, and contains nothing of special interest, except that he had been seriously ill. During his illness the asylum was managed by Dr. Kerr, the Senior Assistant Medical Officer. The maintenance charge was 8s. 9d. per head per week.

### JOINT COUNTIES' ASYLUM, CARMARTHEN.

At the beginning of the year this asylum had 650 patients in residence, and at the close of the year the numbers had fallen to 633. There were 102 first admissions, 14 readmissions, 29 recoveries, 7 discharged relieved, and 76 deaths. The average number resident was 633, and the total number under treatment was 763. Calculated on the admissions the recoveries stand at 29·8 per cent., and calculated on the average number resident the deaths stand at 12 per cent.—the former percentage is a low one and the latter a high one. Of the deaths 22 were caused by cerebral and spinal diseases, 14 by consumption, 13 by pneumonia, 5 by bronchitis, and 4 by general tuberculosis. Of the year's admissions 16 owed the insanity to domestic trouble, mental anxiety, or adverse circumstances; 12 to intemperance in drink, 20 to hereditary influence, and 13 to congenital defect. In analysing the admissions of the year Dr. Goodall mentions the cases of two brothers with the following family history:—"The paternal uncle had been an inmate of the asylum; the patient's parents were first cousins; their mother, and two brothers, and paternal grandfather died of consumption; two brothers are deaf and dumb. Since as much as this was elicited, there was doubtless more to illustrate the degenerate state of this family." These cases are by no means isolated ones. On the contrary, parallels might be found in every county asylum in England, and we should search in vain for any steps being taken to prevent their recurrence in the next generation. It would seem as if vast sums were being spent with the object of curing a few more patients, and not one farthing on the much more important point of preventing insanity. Even in this report we read that the "pathological laboratory work has been consistently carried out, and the results communicated to certain of the medical journals." The asylum is pretty nearly full, but there are 43 out-county patients who could be discharged if room were wanted for in-county cases. The visiting committee "speak in the highest terms of the medical superintendent, who performs his duties with great ability and tact." The weekly rate of maintenance is 7s. 7d. per head.

